# A High-Throughput Behavioral Assay for Screening Novel Anxiolytics in Larval Zebrafish

**DOI:** 10.3390/ph18070968

**Published:** 2025-06-27

**Authors:** Alice Dang, Gina Zhao, Jiale Xu, Mahendra Wagle, Su Guo

**Affiliations:** 1 Department of Bioengineering and Therapeutic Sciences, University of California, San Francisco, CA 94143-2811, USA; 2 Graduate Programs of Pharmaceutical Sciences and Pharmacogenomics, Neuroscience, Tetrad, Developmental and Stem Cell Biology, University of California, San Francisco, CA 94143, USA; 3 Institute of Human Genetics, University of California, San Francisco, CA 94143, USA; 4 Kavli Institute of Fundamental Neuroscience, University of California, San Francisco, CA 94143, USA; 5 The Eli and Edythe Broad Center of Regeneration Medicine and Stem Cell Research, University of California, San Francisco, CA 94143, USA; 6 Bakar Aging Research Institute, University of California, San Francisco, CA 94143, USA

**Keywords:** anxiety, zebrafish, high-throughput screening, strong dark avoidance, anxiolytics, behavioral assay

## Abstract

**Background:** Anxiety disorders affect millions of people worldwide, but current treatments often have limited effectiveness and produce unpredictable responses in patients. This underscores the need for novel anxiolytics. While behavior-based screens are valuable for discovering new small molecules, high-throughput anxiety-related assays in vertebrates are lacking. The larval zebrafish dark avoidance, which corresponds to aversive responses to a predator’s shadow, can be reduced by known anxiolytics and involves neural pathways known to regulate human anxiety. **Methods:** Larval zebrafish exhibiting strong dark avoidance (SDA) have been characterized, reflecting pathological anxiety. We developed a high-throughput behavioral assay using a 96-well plate and showed that SDA larvae displayed significant dark avoidance in this setup. We tested known anxiolytics, Chlordiazepoxide and Buspirone, and found that they significantly reduced dark avoidance in this 96-well assay. We then tested a new candidate compound, Analgesic Screen 1 (AS1), previously shown to reverse avoidance of noxious stimuli such as high temperature in larval zebrafish. **Results:** The optimized 96-well plate assay reliably detected the anxiolytic activity of Chlordiazepoxide and Buspirone and revealed the effect of AS1 in reducing dark avoidance, thereby establishing the platform’s sensitivity and validity. **Conclusions:** This study demonstrates that the dark avoidance assay is scalable to a 96-well plate format in a small arena. This finding provides an effective platform for discovering novel anxiolytic compounds.

## 1. Introduction

Anxiety in response to perceived threats is an evolutionarily conserved stress response essential for survival, but its dysregulation can result in debilitating psychiatric disorders, including generalized anxiety disorder (GAD), panic disorder, and phobias, which collectively represent a major public health challenge by severely impacting daily life, productivity, and well-being [1]. Abnormal anxiety also shows extensive comorbidity with other mental disorders such as depression, schizophrenia, and substance use disorders. Despite the availability of various classes of anxiolytic medications, current treatments are limited by modest efficacy, delayed onset of action, and undesirable side effects [1,2,3].

Pharmacological management of anxiety disorders primarily includes benzodiazepines (BZDs), selective serotonin reuptake inhibitors (SSRIs), serotonin-norepinephrine reuptake inhibitors (SNRIs), Buspirone, and certain anticonvulsants [4,5,6]. BZDs act as positive allosteric modulators at GABA_A_ receptors, rapidly reducing anxiety but are associated with sedation, psychomotor impairment, tolerance, and dependence with chronic use [4]. SSRIs/SNRIs are first-line agents as they enhance serotonergic and noradrenergic neurotransmission, though their anxiolytic effects are delayed, and side effects such as lack of appetite, gastrointestinal upset, and sexual dysfunction often limit adherence [5]. Buspirone, a partial 5-HT_1_A agonist, and certain antiepileptics and atypical antipsychotics are alternatives for refractory cases [6]. The discovery of first-in-class anxiolytics has historically stemmed from serendipitous phenotypic observations at the behavioral levels in animals and humans. Recent decades have seen increased rational drug design based on advances in biology and chemistry [3]. Despite these strides, treatment resistance and suboptimal therapeutic responses remain widespread, highlighting the urgent need for better translational models and targeted therapies [3].

Animal models have been instrumental in the preclinical discovery and validation of anxiolytic compounds and in elucidating the underlying neurobiological mechanisms of anxiety [7]. Traditional rodent assays, such as the elevated plus maze, open field, and light/dark box, demonstrate strong face validity (behaviors resembling human anxiety), predictive validity (response to clinically effective anxiolytics), and some construct validity (mimicking brain circuit and neurotransmitter involvement) [8]. However, these mammalian models are constrained by high costs, lower throughput, and lower suitability for early-stage, large-scale screening. There are also limitations in their capacity to recapitulate the pathological states seen in human anxiety-related disorders [9].

Zebrafish (*Danio rerio*) have recently emerged as an invaluable alternative model for behavioral neuroscience and drug discovery. Their rapid development, genetic tractability, and transparency in the larval stage enable high-throughput assays and real-time phenotyping [10,11]. Approximately 70% of human genes have a zebrafish ortholog and their neural circuits and major neurotransmitter systems, including GABAergic, serotonergic, dopaminergic, and glutamatergic pathways, are highly conserved and amenable to pharmacological interrogation [12,13]. Zebrafish behavioral paradigms have now been validated for a range of neuropsychiatric phenotypes, including anxiety, and are recognized for their translational relevance and utility in drug screening pipelines [14,15]. Recent reviews and comparative studies support the use of zebrafish as a predictive and efficient platform for neuropsychiatric drug discovery [16,17,18].

The light/dark preference assay is one of the best-validated zebrafish models for anxiety research. Larval zebrafish show an innate aversion to dark environments, and this behavior involves a complex neural circuitry [19,20]. Furthermore, this behavior can be reliably attenuated by known anxiolytics, including SSRIs, GABA_A_ agonists (e.g., benzodiazepines), and serotonergic agents [21,22,23]. Heritable variations in this dark avoidance have been documented, with some larvae exhibiting strong dark avoidance (SDA), potentially modeling pathological anxiety states [24]. This natural variation increases assay sensitivity and provides a unique opportunity to characterize both genetic and pharmacological modifiers of anxiety-like behavior. The light/dark preference test demonstrates face, construct, and predictive validity in its ability to replicate aspects of anxiety and respond to known anxiolytics.

In this study, we build upon these findings to develop and optimize a high-throughput light/dark preference assay in the 96-well plate using SDA zebrafish larvae, enabling efficient screening of small molecule libraries for anxiolytic activity. Our goals were to: (1) establish and validate this high-throughput behavioral assay and (2) demonstrate its reliability and sensitivity by testing known anxiolytics and evaluating a novel candidate anxiolytic, as proof-of-concept [25]. This approach leverages the unique strengths of the zebrafish model to accelerate the early-phase discovery of potential therapeutics for anxiety disorders.

## 2. Results

### 2.1. Development of a Light-Dark Preference Assay in the 96-Well Format

To enable high-throughput behavioral screening for anxiety-modulating compounds in zebrafish larvae, we systematically developed and optimized a light-dark preference assay suitable for the 96-well plate format. The principal aim was to maximize throughput, minimize compound usage, and ensure reliable, automated tracking of larval behavior. The optimization process involved evaluating several assay formats and technical approaches.

**Figure 1:**
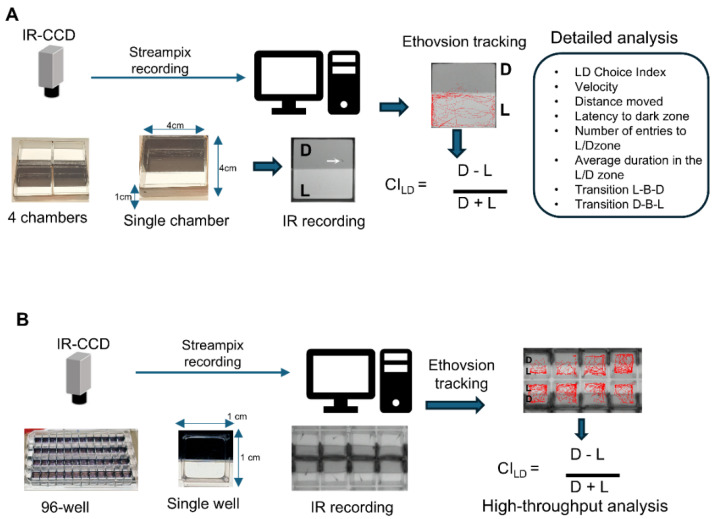
Comparison of the conventional light-dark preference assay vs the 96-well plate assay. (**A**) Schematic of light/dark choice behavior assay carried out in conventional 4 cm × 4 cm behavior chambers, allowing detailed analysis of the behavior. (**B**) Schematic of light/dark choice behavior assay carried out in a customized 96-well plate for high-throughput analyses.

Our earliest design utilized large custom chambers of 4 cm × 4 cm × 1 cm (L × W × H) with a 10 mL liquid holding capacity (Figure 1A). This setup has a clear separation of light and dark zones with sufficient space for larvae to explore, allowing more complex parameters to be analyzed such as latency to dark zone, number of visits to each zone, transition to the other zone after approaching the boundary [24]. However, it suffered from disadvantages such as a requirement for high volumes of testing compounds per replicate. Additionally, it posed considerable challenges for high-throughput screening, both logistically and in terms of resource consumption. Only 16 behavioral chambers could be recorded with a single camera. This combination of high material usage and limited high-throughput capacity made it clear that a more scalable and resource-efficient platform was necessary for effective compound screening.

Subsequent iterations reduced the chamber volume to 4 mL and later to 2 mL using square wells. These versions decreased the required volume of compounds and improved handling. However, the computer tracking system experienced difficulties in accurately following larvae, particularly when they approached the edges or corners of the chambers due to the height of the walls. This loss of resolution interfered with reliable behavioral quantification.

We also searched for efficient ways to establish physically distinct light and dark areas within each test chamber. One approach employed a computer monitor placed beneath transparent plates, which displayed alternating light and dark regions in 4-min intervals over a total of 20 min. However, this method produced video recordings of insufficient quality, with excessive graininess and artifacts that further compromised automated tracking accuracy.

The final, optimized assay used a commercially available 96-well plate with square wells (500 μL liquid holding capacity per well), positioned atop a white LED light box. To reliably generate distinct light and dark zones beneath each well, we placed custom-cut black and clear acrylic strips directly underneath the plate (Figure 1B). This physical barrier provided a sharp, reproducible contrast between dark and light regions within each well. However, we found that this setup alone was insufficient: when only acrylic strips were used, larvae frequently failed to show the expected strong dark avoidance (SDA) behavior. This observation suggested that zebrafish larvae could still perceive light leakage around the edges of each well, effectively reducing the contrast between the supposed dark and light zones.

To address this, we outlined the inside walls of each well corresponding to the dark zone with an opaque black Sharpie marker. This additional step significantly enhanced the separation between the light and dark compartments by eliminating peripheral light bleeding from the light box, thereby preventing larvae from seeing past the bottom zone divisions and ensuring robust, reliable measurement of SDA behavior. The resulting configuration produced distinct visual zones within each well and was essential for consistently eliciting and quantifying the desired behavioral response. Behavioral recording was accomplished via a four-camera setup equipped with infrared filters and images were captured and analyzed using the Streampix 9.3.1.270(x64) software.

This configuration achieved several critical goals: it substantially minimized the amount of compound required per assay, was compatible with high-throughput screening, and, importantly, enabled precise, automated tracking of larval zebrafish within individual wells. The system provided robust, reproducible partitioning of light and dark areas and produced high-quality video output suitable for downstream behavioral analysis.

### 2.2. Effects of Known Anxiolytics in the 96-Well Light-Dark Preference Assay

**Figure 2:**
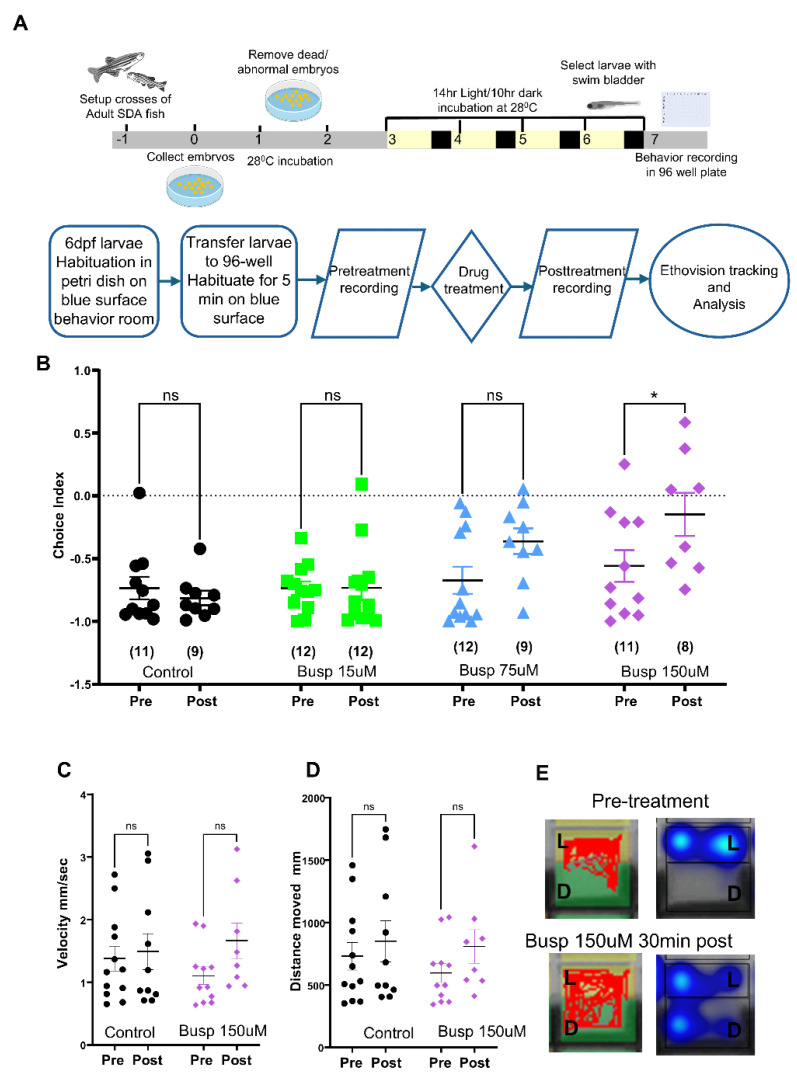
Validation of 96-well light/dark choice behavior assay with known anxiolytic Buspirone: (**A**) schematic showing larvae raising and preparation for the 96-well plate light/dark behavioral assay. (**B**) Scatter plot showing light/dark choice index comparisons of pre and post-treatment groups, numbers in parentheses indicate the number of larvae corresponding to the treatment group. (**C**) Scatter plot for velocity and (**D**) distance moved by larvae during the recording. Mixed-effects model (REML) and Šídák’s multiple comparisons test, * *p* < 0.05, ns—non significant. (**E**) Example of tracks and heatmap.

The light/dark preference test is an established model for studying anxiety-like behavior in zebrafish. This assay measures the natural light/dark preference behavior, which can be modified by anxiolytic compounds. To validate our high-throughput screening platform’s capability to detect anxiolytic effects, we utilized anxiolytics Chlordiazepoxide and Buspirone that are known to reduce dark avoidance in larval zebrafish [23]. Based on our earlier studies, we tested various concentrations and durations of treatment to find the optimal conditions. We tested Buspirone at 15 uM, 75 uM, and 150 uM for 30 min, 1 h, and 1.5 h. Bath application of Buspirone at a 150 uM final concentration for 30 min showed a significant reduction in dark avoidance compared to the pre-treatment (CI_LD_ ± SEM n and *p* values for Control pre −0.737 ± 0.089 n = 11 post −0.816 ± 0.057 n = 8, *p* = 0.9743 and for Buspirone pre −0.56 ± 0.12 n = 11, post −0.15 ± 0.17 n = 8, *p* = 0.386 mixed-effects model (REML) and Šídák’s multiple comparisons test, Figure 2B).

Similarly, Chlordiazepoxide was tested at 20 uM, 100 uM, and 200 uM for 30 min, 1 h, and 1.5 h. The Chlordiazepoxide treatment at 200 uM for 30 min resulted in a significant reduction in dark avoidance (CI_LD_ ± SEM n and *p* values for Control pre −0.824 ± 0.06 n = 12 post −0.797 ± 0.071 n = 11, *p* = 0.999 and for 200 uM Chlordiazepoxide pre −0.69 ± 0.073 n = 11 post −0.247 ± 0.071 n = 11 *p* = 0.005 mixed-effects model (REML) and Šídák’s multiple comparisons test, Figure 2A).

**Figure 3:**
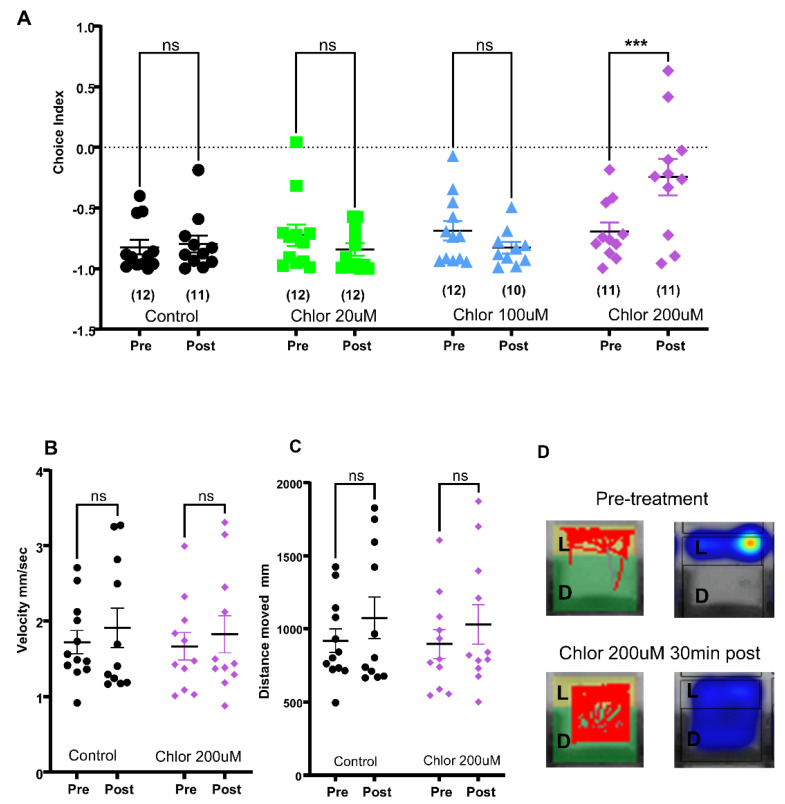
Validation of 96-well light/dark choice behavior assay with known anxiolytic Chlordiazepoxide: (**A**) scatter plot showing light/dark choice index comparisons of pre and post-treatment groups, numbers in parentheses indicate the number of larvae corresponding to the treatment group. (**B**) Scatter plot for velocity and (**C**) distance moved by larvae during the recording. Mixed-effects model (REML) and Šídák’s multiple comparisons test, *** *p* < 0.001, ns—non significant. (**D**) example of tracks and heatmap.

Both compounds did not significantly affect the velocity and distance moved by the larvae, indicating the motor activity of larvae was not affected by the anxiolytic treatment (Figure 2C–E and Figure 3B–D). Thus, our 96-well light/dark choice behavior assay provides a valid setup to identify anxiolytics in a high-throughput screen.

### 2.3. Discovery of a Novel Compound, AS1, for Its Anxiolytic Effects Using the 96-Well Light-Dark Preference Assay

**Figure 4:**
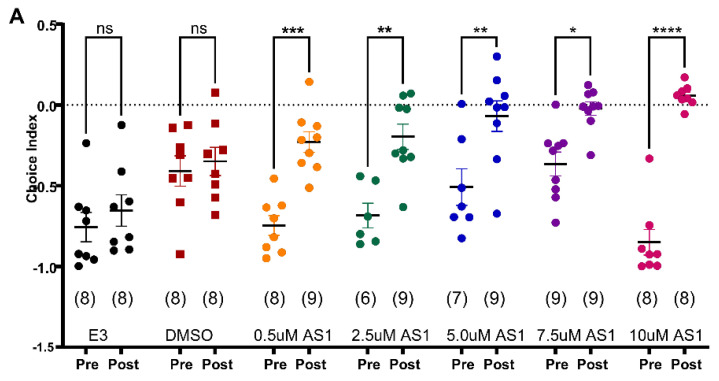

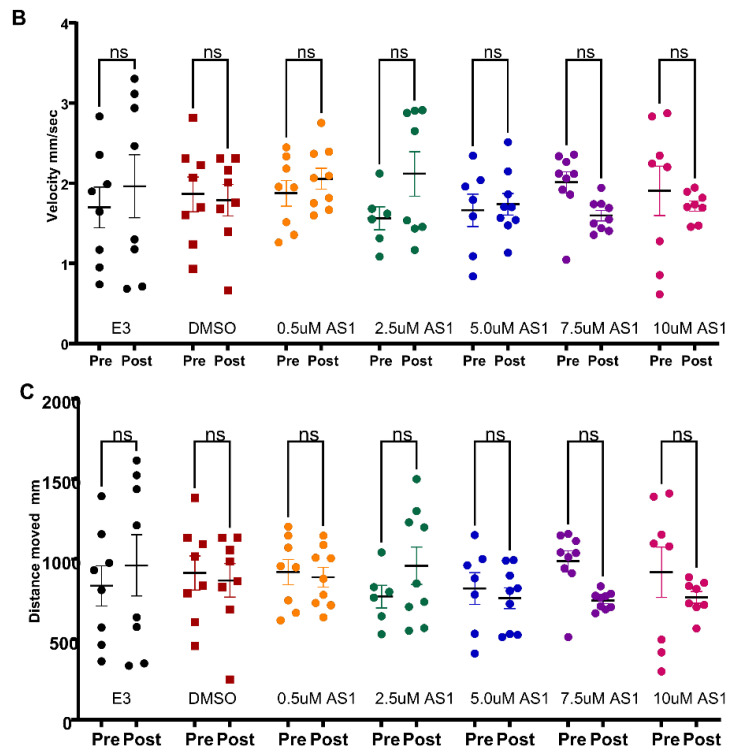
Effect of the new compound AS1 on LD choice behavior in 96-well plate assay. (**A**) Scatter plot showing light/dark choice index comparisons of pre and post-treatment groups, numbers in parentheses indicate the number of larvae corresponding to the treatment group. (**B**) Scatter plot for velocity and (**C**) distance moved by larvae during the recording. Mixed-effects model (REML) and Šídák’s multiple comparisons test, * *p* < 0.05, ** *p* < 0.01, *** *p* < 0.001, **** *p* < 0.0001, ns—non-significant.

AS1 (4-propan-2-yl-N-pyridin-4-ylbenzamide) is a novel small molecule recently identified for its potential to modulate emotional and affective responses. AS1 was first discovered in a screen for compounds that could reverse the negative hedonic valence caused by noxious (painful or aversive) stimuli in zebrafish larvae [23]. We tested AS1 in our 96-well light/dark choice behavior assay. Similar to E3 medium, the bath application of DMSO to a final concentration of 0.1% did not significantly alter the choice index after 1 h of treatment (CI_LD_ ± SEM n and *p* values for 0.1%DMSO pre −0.409 ± 0.093 post −0.35 ± 0.088; *p* = 0.997, mixed-effects model (REML) and Šídák’s multiple comparisons test, Figure 4A). We tested varying concentrations of AS1, ranging from 0.5 uM to 10 uM. At all the tested concentrations, AS1 showed a significant shift in choice index, indicating a reduction in dark avoidance (Figure 4A). At the lowest concentration of 0.5 uM, the choice index for pre –0.747 ± 0.061 n = 8, shifted to post −0.23 ± 0.063 n = 9 with *p* = 0.0007, whereas at the highest 10 uM concentration, the choice index for pre −0.85 ± 0.08 n = 8 shifted to post 0.056 ± 0.024 n = 8 with *p* < 0.0001. We also checked the velocity and distance moved by larvae for all the treatment groups, but did not find any significant differences (Figure 4B,C). Though the velocity and distance moved showed a slight reduction with higher concentrations of 7.5 uM and 10 uM, it was not statistically significant. These results demonstrate our screening platform’s sensitivity in detecting anxiolytic effects, as evidenced by the reduction in dark avoidance behavior following AS1 treatment, validating the assay’s reliability for identifying compounds with anxiety-modulating properties.

## 3. Discussion

Cell-based high-throughput screens (HTSs) have been used for drug discovery, but may lead to compounds unsuitable for in vivo use [26]. A non-vertebrate model system, such as *Caenorhabditis elegans*, was used for small-molecule screens targeting specific genetic pathways [27]. Such high-throughput screens are challenging with mammalian model organisms. Based on the genetic and anatomical similarities with humans, larval zebrafish have emerged as an ideal model system for HTSs [28,29].

Our study demonstrates the successful development and validation of a high-throughput platform for identifying novel anxiolytic compounds using the zebrafish light/dark preference assay. The initial validation of our system using known anxiolytics demonstrated its efficacy in detecting anxiety-modulating effects. This aligns with previous research indicating that anxiolytics can mitigate fear responses in zebrafish behavioral assays [23]. This validation was particularly important as zebrafish have become an advantageous choice in the biomedical field due to their rapid development and suitability for high-throughput screening of both genetic and pharmacological interventions [18,19].

The successful implementation of our 96-well format represents a significant advancement in assay scalability compared to traditional chamber-based assays. By maintaining the fundamental aspects of the validated light/dark preference test while increasing throughput, we have created a robust platform for drug discovery. The clear effects observed with AS1 demonstrate the assay’s sensitivity and reliability in detecting anxiolytic properties of a novel compound. The mechanism by which AS1 affects the dark avoidance behavior is not known. AS1 has been proposed to act via D1 dopamine receptor pathways to elicit an attraction toward noxious stimuli [25]. Further investigation into AS1 targets, genetic manipulation of candidate genes, and brain-wide activity imaging in AS1-treated larvae could help reveal the underlying mechanisms. Our findings lay an important foundation for future studies investigating anxiety mechanisms and screening for novel therapeutics. The combination of our high-throughput platform with AI-assisted analysis could accelerate the discovery of new anxiolytic compounds, potentially leading to more effective treatments for anxiety disorders. This is particularly significant given the current limitations of available anxiety treatments and the need for more targeted therapeutic approaches.

## 4. Materials and Methods

### 4.1. Animals and Housing

All animal protocols in this study were approved by IACUC, University of California, San Francisco (protocol number -AN206836, approved on 22 April 2025). Zebrafish embryos were collected from mass breeding of SDA adult fish [30]. Embryos were transferred to 100 mm Petri dishes containing E3 medium (5 mM NaCl, 0.17 mM KCl, 0.33 mM CaCl_2_ and 0.33 mM MgSO_4_), with 50 embryos per dish, and maintained in a 28 °C incubator. Twenty-four hours post-fertilization, embryos underwent E3 medium rinses and were transferred to 100 mm Petri dishes containing E3 medium. On the third day, hatched larvae were transferred to a blue surface under a 14 h/10 h light/dark cycle to ensure proper vision development (Figure 2A).

### 4.2. High-Throughput Light/Dark Preference Assay Development

The behavioral apparatus was optimized for high-throughput screening using a 96-well plate (Global Life Sciences Solutions USA, LLC., Wilmington, DE, USA, Cat 7701-1651 square wells, 500 μL capacity) with half walls of each well painted with black Sharpie and positioned on a customized trans-illuminator equipped with white LEDs (Cat HitLights Cat # L0512V-402-1630-U). Laser-cut black and clear acrylic strips prepared from transparent and IR transmissible cast acrylic sheets (McMaster Carr, Santa Fe Springs, CA, USA, Cat# 8560K172) and Eplastic Inc., San Diego, CA, USA, Cat ACRY31430.125PM11.555x11.850) were placed beneath the plate to create distinct dark and light zones for each well (Figure 1B). Behavioral recordings were captured using a camera (Camera- Basler acA2040-120 um with lens—Computar M6Z1212-3S 12.5–75 mm, 2/3”, f/1.2, C-Mount) set up with infrared filters prepared from IR transmissible acrylic and connected to a PC with a USB capture card. Behavior videos were captured at 25 fps with 2024 × 1526 pixel resolution for 8 min with Streampix 9.3.1.270(x64) (NorPix Inc. Montréal, QC, Canada) software. The development cost of the setup was under USD10000.

### 4.3. Drug Treatment

The larvae preparation and drug treatment followed the scheme presented in Figure 2A. After habituation in the behavior room, larvae were transferred to 96-well plates with 450 uL of E3 and left on blue background for 5 min to habituate. All plates were transferred to the transilluminator with black and white strips placed on top. The outer frame assured the position and alignment of each well row with black and transparent strips. After the 8 min of pre-treatment recording, plates were moved back on to the blue background. Next, 50 uL of 10× concentrated drug or vehicle solution was transferred to each well with multichannel pipette. Plates were left on a shaker at minimal speed for 5 min for mixing the drug solution and returned to the blue background for incubation. Afterwards, the drug treatment plates were placed on a transilluminator and recoded again. For multiple time-point recordings, plates were kept on a blue background in between the recordings.

### 4.4. Behavioral Analysis

Behavioral analysis was conducted using Ethovision XT software version 13 (Noldus, Leesburg, VA, USA to track movement parameters including duration in light and dark zones. The quantitative data from the Ethovisoin was exported to MS Excel files. Data from the larvae that were tracked for at least 65% of the recording time was used for further analysis. The light/dark choice index (CI_LD_) was calculated as (time in dark zone—time in light zone)/(time in dark zone + time in light zone), where values range from +1 (complete light aversion) to −1 (complete dark aversion).

### 4.5. Statistical Analysis

Data analysis was performed using GraphPad Prism 10 software, employing mixed-effects model (REML) and Šídák’s multiple comparisons test for multiple group comparisons.

## Data Availability

The data presented in the study will be available upon request to the corresponding authors. The data are not publicly available as it is stored on a secure server.

## Abbreviations

The following abbreviations are used in this manuscript:

SDA	Strong dark avoidance.
GAD	Generalized anxiety disorder
BZDs	Benzodiazepines
SSRIs	Selective serotonin reuptake inhibitors
SNRIs	Serotonin-norepinephrine reuptake inhibitors
5-HT	5-hydroxytryptamine, serotonin
GABA	Gamma-aminobutyric acid
CI_LD_	Light/dark choice index
